# The correlation between tumor radiological features and spread through air spaces in peripheral stage IA lung adenocarcinoma: a propensity score-matched analysis

**DOI:** 10.1186/s13019-024-02498-0

**Published:** 2024-01-23

**Authors:** Chao Jia, Hai-Cheng Jiang, Cong Liu, Yu-Feng Wang, Hong-Ying Zhao, Qiang Wang, Xiu-Qing Xue, Xiao-Feng Li

**Affiliations:** 1 Department of Radiology, The Xuzhou Hospital Affiliated to Jiangsu University, Xu Zhou, Jiang Su 221004 People’s Republic of China; 2 Department of Thoracic Surgery, Xuzhou Cancer Hospital, Xuzhou, 221000 People’s Republic of China; 3 Department of Puncture Minimally Invasive, Xuzhou New Health Hospital, Xuzhou, 221000 People’s Republic of China; 4 Department of Minimally Invasive Oncology, Xuzhou New Health Hospital, Xuzhou, 221000 People’s Republic of China; 5 Department of Nuclear Medicine, Xuzhou Cancer Hospital, Xuzhou, 221000 People’s Republic of China; 6 Department of Medical Oncology, Xuzhou Cancer Hospital, Xuzhou, 221000 People’s Republic of China; 7 Department of Radiotherapy, Xuzhou Cancer Hospital, Xuzhou, 221000 People’s Republic of China; 8 Department of Nuclear Medicine, Yancheng First Hospital, Affiliated Hospital of Nanjing University Medical School, The First People’s Hospital of Yancheng, Yancheng, 224005 People’s Republic of China; 9 Department of Radiology, Xuzhou Cancer Hospital, Xuzhou, 221000 People’s Republic of China

**Keywords:** Adenocarcinoma, Consolidation tumor ratio, Tumor spread through air spaces, Odds ratio

## Abstract

**Background:**

The consolidation tumor ratio (CTR) is a predictor of invasiveness in peripheral T1N0M0 lung adenocarcinoma. However, its association with spread through air spaces (STAS) remains largely unexplored. We aimed to explore the correlation between the CTR of primary tumors and STAS in peripheral T1N0M0 lung adenocarcinoma.

**Methods:**

We collected data from patients who underwent surgery for malignant lung neoplasms between January and November 2022. Univariate and multivariate analyses following propensity-score matching with sex, age, BMI, were performed to identify the independent risk factors for STAS. The incidence of STAS was compared based on pulmonary nodule type. A smooth fitting curve between CTR and STAS was produced by the generalized additive model (GAM) and a multiple regression model was established using CTR and STAS to determine the dose-response relationship and calculate the odds ratio (OR) and 95% confidence interval (CI).

**Results:**

17 (14.5%) were diagnosed with STAS. The univariate analysis demonstrated that the history of the diabetes, size of solid components, spiculation, pleural indentation, pulmonary nodule type, consolidation/tumor ratio of the primary tumor were statistically significant between the STAS-positive and STAS-negative groups following propensity-score matching(*p* = 0.047, 0.049, 0.030, 0.006, 0.026, and < 0.001, respectively), and multivariate analysis showed that the pleural indentation was independent risk factors for STAS (with *p*-value and 95% CI of 0.043, (8.543–68.222)). Moreover, the incidence of STAS in the partially solid nodule was significantly different from that in the solid nodule and ground-glass nodule (Pearson Chi-Square = 7.49, *p* = 0.024). Finally, the smooth fitting curve showed that CTR tended to be linearly associated with STAS by GAM, and the multivariate regression model based on CTR showed an OR value of 1.24 and a p-value of 0.015.

**Conclusions:**

In peripheral stage IA lung adenocarcinoma, the risk of STAS was increased with the solid component of the primary tumor. The pleural indentation of the primary tumor could be used as a predictor in evaluating the risk of the STAS.

## Background

Multiple clinical studies have shown that screening with low-dose computed tomography (LDCT) can lower the mortality rate of lung cancer and improve prognosis [1–3]. With the widespread use of LDCT for lung cancer screening in high-risk populations, the incidence of early-stage lung cancer is gradually increasing. LDCT has a detection rate of about 3.48% for lung cancer, with 81.09% of cases being stage I cancer [3]. Non-small-cell lung cancer (NSCLC) comprises 80–85% of all types of lung cancer [4, 5], with adenocarcinoma being the predominant pathological subtype, accounting for approximately 40% [6]. For stage IA lung adenocarcinoma, radical resection is still the preferred and recommended treatment according to guidelines [7, 8].

In 2020, Yasuhiro [9] has proposed that sublobar resection for stage IA lung adenocarcinoma can achieve a prognosis that is not inferior to that of lobectomy. However, the heterogeneity of malignant tumors still leads to differences in long-term prognosis after surgery [10]. Suzuki [11] has suggested that sublobar resection may be more suitable for patients with less-invasiveness stage IA lung adenocarcinoma. Therefore, recognizing less-invasiveness stage IA lung adenocarcinoma preoperative has become a significant challenge for thoracic surgeons [12]. Our previous research has confirmed that sublobar resection is more suitable for patients with less-invasiveness stage IA lung adenocarcinoma based on the metabolic parameters of the primary tumor [13]. In recent years, spread through air spaces (STAS) as a manifestation of early lung cancer has received increasing attention from clinical researchers [12, 14]. Previous studies [14] have shown that STAS can increase the recurrence rate after limited resection for stage IA lung adenocarcinoma, making it an essential feature of the invasion of stage IA lung adenocarcinoma. Consolidation tumor ratio (CTR) is defined as the ratio of the maximum size of a solid component to the maximum tumor size in the primary tumor and can be used to predict the invasion of peripheral stage IA lung adenocarcinoma lesions. Moreover, according to Suzuki [11], CTR ≤ 0.25 can serve as a less invasive means of identifying lung adenocarcinoma lesions in the peripheral stage IA.

Currently, there are many clinical studies about CTR with STAS [15, 16], previous study confirmed that the CTR of primary tumor was association with STAS-positive tumors [15, 17]. However, it is still lack of the clinical evidence of CTR with STAS in peripheral stage IA lung adenocarcinoma. We aimed to investigate the correlation between the primary tumor CTR and STAS in patients with peripheral stage IA lung adenocarcinoma.

## Methods

### Study population and data collection

This study was a retrospective analysis of data from consecutive malignant lung neoplasm patients who underwent surgical resection at Xuzhou Hospital affiliated with Jiangsu University between January 1, 2022, and November 30, 2022. The study enrolled patients who fulfilled the following inclusion criteria: (1) having a single tumor, (2) the peripheral lesion located in the outer third of the lung field on chest CT axial image, with the tumor’s center in this region [18], (3) having a maximum diameter of the primary tumor on chest CT lung window axial image ≤ 3 cm, and not having any lymph nodes with the shortest diameter ≥ 10 mm observed on the mediastinal window, and (4) having a pathological diagnosis of adenocarcinoma after undergoing surgery. Patients were not eligible for the study if they met any of the following criteria: (1) removal of fewer than six lymph nodes during surgery [19], (2) lack of 0.625-1 mm thin-layer imaging or incomplete imaging or clinical data on preoperative chest CT examination, (3) receiving prior anti-tumor therapy, such as radiotherapy, chemotherapy, targeted therapy, or immunotherapy, before surgery, (4) a history of malignant tumors in the previous 3 years, (5) suspected tumor metastasis on preoperative examination, or (6) an interval of more than one week between the CT examination and surgery. The primary endpoint of the study was tumor STAS, as diagnosed by postoperative pathology. The presence of tumor cells within air spaces in the lung parenchyma beyond the edge of the main carcinoma [14] was defined as STAS positive. We collected the information from the patients’ medical records, including their sex, age, BMI, smoking status, hypertension, diabetes, coronary heart disease, family history, and history of pulmonary disease. The patients’ history of pulmonary disease included chronic obstructive pulmonary disease (COPD), pulmonary fibrosis, emphysema, bullae, chronic inflammation, and bronchiectasis. The smoking status was recorded as “never” for individuals who had never smoked or had quit smoking for more than 10 years, and as “ever” for all other conditions. We conducted the study in accordance with the Declaration of Helsinki (as revised in 2013), The ethics committee approved the study protocol, and due to the retrospective nature of our study, informed consent from patients was waived.

### Image evaluation and data measurement

**Fig. 1:**
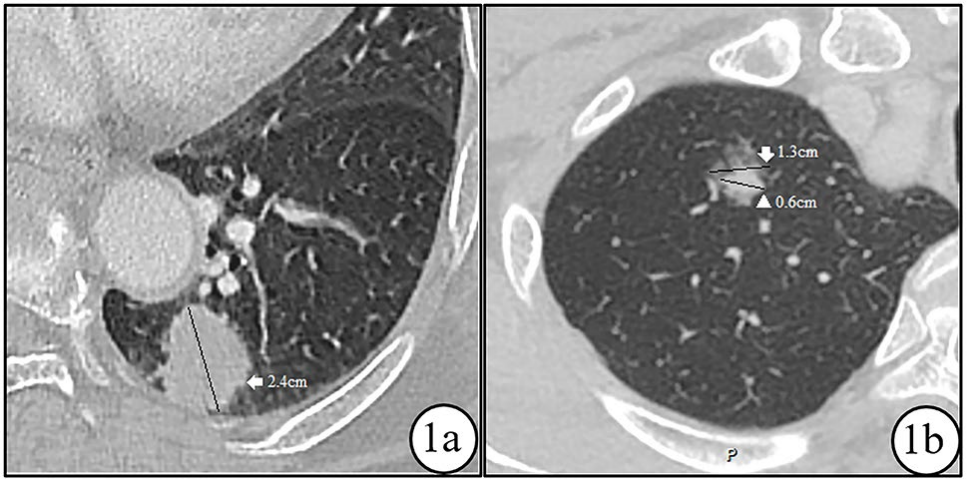
**a** shows a female patient aged 58 with a solid nodule in the lower lobe of the left lung, as seen on CT scan with a maximum diameter of 2.4 cm (indicated by the white arrow). The nodule has a CTR of 1. Similarly, in **b**, a male patient aged 61 is shown to have a partially solid nodule in the upper lobe of the right lung, with a solid component size of 0.6 cm (indicated by the white arrowhead) and a maximum diameter of 1.3 cm (indicated by the white arrow). The CTR for this lesion is 0.6/1.3 = 0.46

**Fig. 2:**
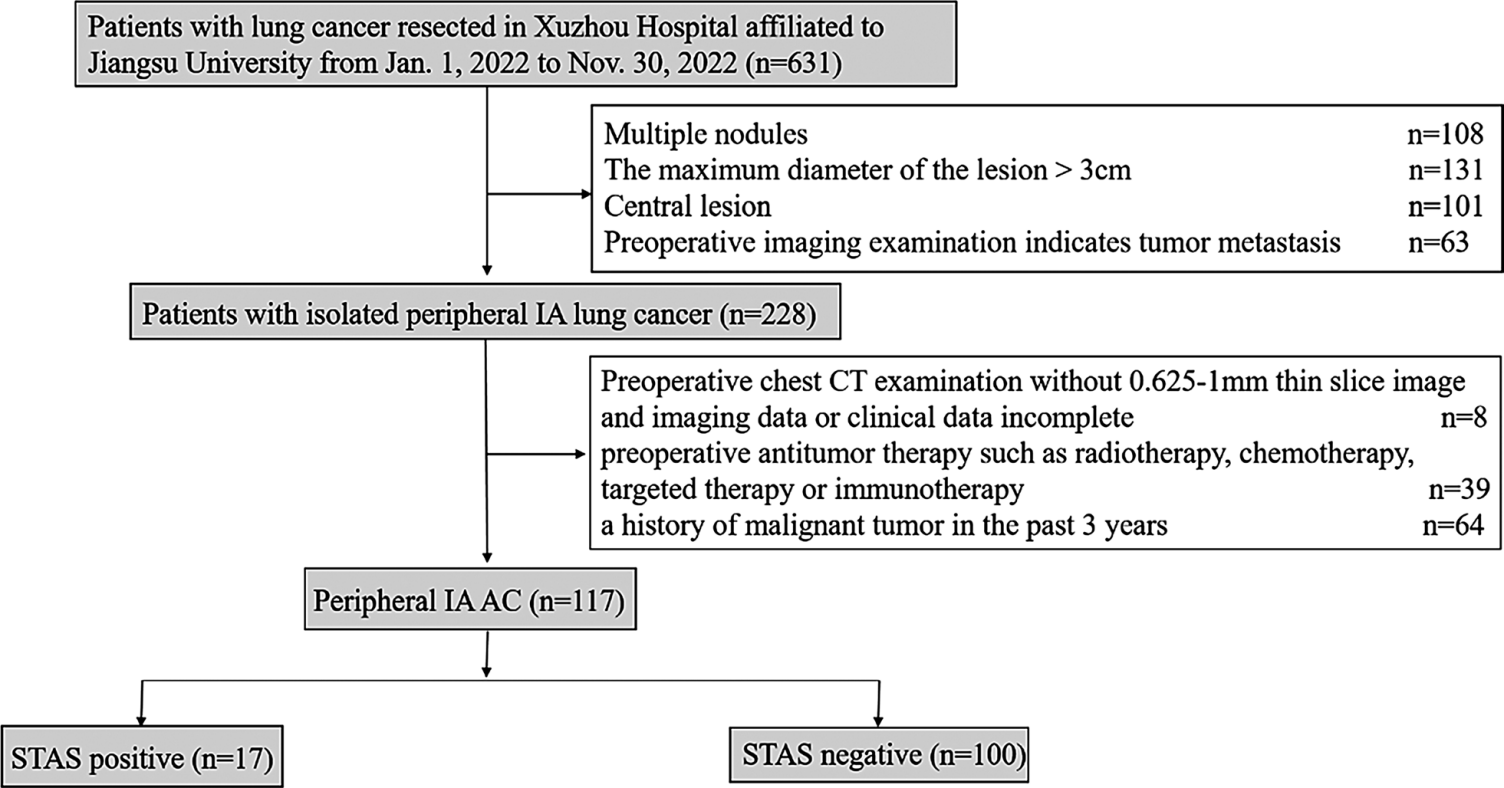
Flow chart of the population selection

Two chest radiologists (C.J, with 11 years of experience in thoracic oncology, and L.L.W, with 5 years of experience in thoracic oncology) evaluated and measured the CTR, and then another senior radiologists evaluated the accuracy of the measurement, and taken the average value of the two measurements. The evaluation and measurement of imaging parameters were carried out based on 1-mm thin-layer CT axial lung window images. The qualitative indicator parameters consisted of location, lobar lung, lobulation, spiculation, vacuole, pleural indentation, internal vascular sign, and bronchial abnormality. Lobulation referred to the presence of multiple arched surfaces on the nodule, resembling petals. Spiculation manifested as radiating, unbranched, straight, and sharp lines extending from the edge of the nodule, with slightly thicker lines near the nodule’s end. Vacuole referred to the translucent area inside the lesion that was less than 5 mm. Pleural indentation was characterized by the shrinkage and traction of fibrous components inside the nodule, forming a triangular or trumpet-shaped water-density area on the visceral pleura without pleural thickening or adhesion. Internal vascular signs were classified into four types [20]: type I referred to vessels passing near the nodule; type II referred to vessels passing through the nodule without changes in size or shape; type III referred to twisted and dilated vessels visible inside the nodule, with no increase in number; and type IV referred to more complex vascular structures, such as vascular malformation [20]. Bronchial abnormality were classified into three types: type I referred to the absence of bronchial branches inside the nodule; type II referred to the presence of bronchial branches inside the nodule with no changes in shape; and type III referred to changes in shape, such as twisting, stiffness, narrowing, or dilation of bronchial branches inside the nodule [20]. The quantitative indicator parameters included (1) the maximum diameter of the nodule, which referred to the largest measured diameter of the nodule in the transverse lung window at the maximum level (Fig. 1a); (2) the maximum diameter of the solid component of the nodule, which referred to the largest measured diameter of the solid component of the nodule in the axial lung window at the maximum level (Fig. 1b); and (3) the CTR, defined as the ratio of the maximum diameter of the solid component of the tumor to the maximum diameter of the tumor in the lung window [11]. Pure ground-glass nodules were recorded as 0, solid nodules were recorded as 1, and the CTR of partially solid nodules was between 0 and 1.

### Statistical analysis methods

For continuous variables with a normal distribution, the results were expressed as the mean ± standard deviation (SD). The median and interquartile range (IQR) was reported for non-normally distributed data. Categorical variables were presented as integers and proportions, and normality was assessed using the Kolmogorov-Smirnov test. The Student’s t-test was used for normally distributed continuous variables, while the Wilcoxon-Mann-Whitney U test was used for non-normally distributed variables to assess differences. The chi-square test or Fisher’s exact test was employed to compare categorical variables. To assess the reproducibility of measurements for quantitative variables, the intraclass correlation coefficient (ICC) [21] was calculated for inter-observer and intra-observer agreement for the maximum diameter of nodules and the maximum diameter of solid components. Thirty randomly selected patients were re-measured, and ICCs were calculated. An ICC > 0.75 indicated good agreement. Propensity score-matched analysis was performed according to sex, age, and BMI at 1:2 for both groups. For patient matching, the nearest neighbor matching method with a caliper of 0.25 was adopted for the continuous variable (age, BMI) and exact matching was adopted for the categorical variables (sex). Statistically significant variables (*p* < 0.05) from the univariate analysis were included in a backward likelihood ratio binary logistic regression analysis. A variance inflation factor (VIF) ≤ 5 was calculated using a simple linear regression model to avoid the collinearity issue. The CTR values were divided into three tertiles based on the pulmonary nodule type, and a trend test was used to assess the covariates related to the tertiles of CTR. Finally, the generalized additive model (GAM) describes the relationship between CTR and STAS by a smooth fitting curve, and a multivariate regression model was constructed to calculate the odds ratio (OR) of CTR in STAS. All statistical analyses were conducted using R software (version 3.4.3, https://www.r-project.org, R packages: glmnet, pROC, rms, and dca. R), with a two-tailed *P* value of less than 0.05 considered statistically significant.

## Results

### Baseline characteristics of the enrolled patients

Among the 117 patients who were finally enrolled in the study, 17 cases of STAS (14.5%) were diagnosed, and Fig. 2 illustrates the patient inclusion flowchart. Additionally, 11 cases of occult lymph node metastasis were observed (9.4%). Table 1 presents the baseline characteristics of the patients in STAS-positive and STAS-negative groups before and after propensity-score matching. There was no statistically significant difference in sex distribution between the both groups (*p* = 0.991). The incidence of STAS was higher in the middle-aged group (< 65 years) and non-smoking group than in the elderly group and smoking group, respectively, but there was no significant difference (*p* = 0.068 and 0.864, respectively). The variables including diabetes, spiculation, pleural indentation, size of solid components, pulmonary nodule type and CTR were significant difference between STAS-positive and STAS-negative groups after propensity-score matching (*p* = 0.047, 0.030, 0.006, 0.049, 0.026 and < 0.001, respectively). Furthermore, although all STAS-positive lesions exhibited bronchial abnormality, lobulations, and internal vascular signs, there was no statistically significant difference between the two groups (*p* = 0.401, 0.100, and 0.679, respectively). The multivariate analysis results demonstrated that the pleural indentation of the primary tumor was independent risk factors for STAS (with *p*-value and 95% CI of 0.043, (8.543–68.222)). Additionally, the maximum nodule diameter and maximum solid component diameter were measured in 30 randomly selected patients, and both intra-observer and inter-observer consistency coefficients were calculated. The results showed that the intra-observer and inter-observer consistency coefficients for the maximum nodule diameter and maximum solid component diameter were 0.994 (0.987–0.997), 0.998 (0.996–0.999), 0.988 (0.975–0.994), and 0.996 (0.992–0.998), respectively. These values were greater than 0.9, indicating good consistency in the measurement of quantitative indicators.

**Table 1:** Baseline characteristics of the enrolled patients before and after propensity-score matching

Variables		Before match (*N* = 117)				After match (*N* = 51)		
		STAS positive	STAS negative	Z/x^2^ value	P-value	STAS positive	STAS negative	P-value
N		17	100			17	34	
Sex, n (%)	Male	9 (52.9)	50 (50.0)	0.05	0.823	9 (52.9)	21 (61.8)	0.763
Age	Median [Min, Max]	61.0 [44.0, 75.0]	61.5 [30.0, 82.0]	−0.147	0.883	61.0 [44.0, 75.0]	64.0 [34.0, 82.0]	0.358
	<65	14 (82.4)	59 (59.0)	3.377	0.066	14 (82.4)	19 (55.9)	0.120
Smoking status, n (%)	Ever	5 (29.4)	31 (31.0)	0.017	0.896	5 (29.4)	14 (41.2)	0.609
BMI	Median [Min, Max]	22.9 [21.5, 25.3]	24.2 [21.4, 26.0]	−0.623	0.534	22.9 [17.9, 32.0]	23.7 [17.6, 29.3]	0.806
	≥ 24	6 (35.5)	53 (53.0)	1.822	0.177	6 (35.3)	17 (50.0)	0.486
Hypertension, n (%)	Positive	2 (11.8)	26 (26.0)	1.617	0.203	2 (11.8)	8 (23.5)	0.533
Diabetes, n (%)	Positive	1 (5.9)	32 (32.0)	4.895	0.027	1 (5.9)	14 (41.2)	**0.023**
CAD, n (%)	Positive	7 (41.2)	42 (42.0)	0.004	0.949	7 (41.2)	17 (50.0)	0.766
Family history, n (%)	Positive	0 (0)	2 (2.0)	0.364	0.546	0 (0)	3 (8.8)	0.528
History of pulmonary disease, n (%)	Positive	14 (82.4)	61 (61.0)	4.239	0.040	14 (82.4)	21 (61.8)	0.241
Longest diameter, cm		1.800, 1.480	1.600, 0.880	−0.821	0.412	1.80 [0.61, 2.80]	1.90 [0.60, 3.00]	0.886
Size of solid components, cm		1.600, 1.790	0.950, 1.800	−2.166	0.030	1.60 [0, 2.80]	0.95 [0, 2.90]	0.175
Lobulation, n (%)	Positive	17 (100.0)	86 (86.0)	2.703	0.100	17 (100)	32 (94.1)	0.799
Spiculation, n (%)	Positive	16 (94.1)	63 (63.0)	6.416	0.011	16 (94.1)	43 (63.2)	**0.030**
Vacuole, n (%)	Positive	11 (64.7)	54 (54.0)	0.674	0.411	11 (64.7)	34 (50.0)	0.415
Pleural indentation, n (%)	Positive	16 (94.1)	60 (60.0)	7.43	0.006	16 (94.1)	37 (54.4)	**0.006**
Internal vascular sign, n (%)	Positive	17 (100.0)	99 (99.0)	0.171	0.679	17 (100)	67 (98.5)	0.996
Bronchial anomaly sign	Positive	17 (100.0)	96 (96.0)	0.704	0.401	17 (100)	66 (97.1)	0.997
Location, n (%)	Right Lung	14 (82.4)	61 (61.0)	2.879	0.090	14 (82.4)	44 (64.7)	0.268
Lobar lung, n (%)	Superior lobe	8 (47.1)	68 (68.0)	2.799	0.094	8 (47.1)	44 (64.7)	0.290
Pulmonary nodule type, n (%)	Solid nodule	13 (76.5)	44 (44.0)	6.132	0.013	13 (76.5)	29 (42.6)	**0.026**
CTR		1.000, 0.110	0.690, 1.000	−2.166	0.030	1.000, 0.110	0.630, 1.000	**< 0.001**

STAS: Spread through air spaces; CAD: Coronary heart disease; CTR: Consolidation/tumor ratio; *p* ≤ 0.05 considered statistically significant

### The comparison of the incidence of STAS in different pulmonary nodule type

**Fig. 3:**
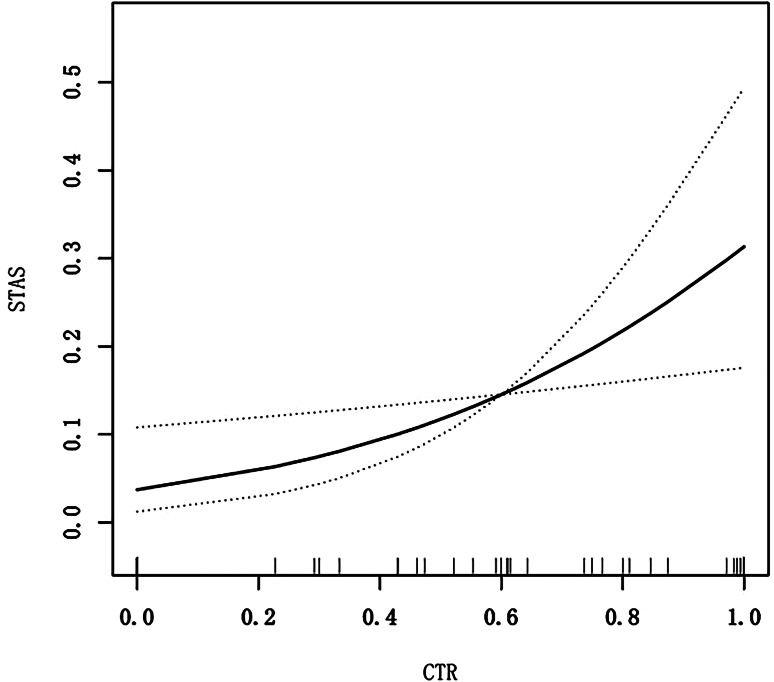
Different types of nodules and microscopic pathological findings. **a**, **b** Male, 64, **a** shows an axial CT of the lung window lesion located in a solid pulmonary nodule in the left lower lobe, with a maximum diameter of 2.5 cm. **b** shows a 100x pathological microscopic image of invasive adenocarcinoma with more dissemination in the STAS. **c**, **d** Male, 61, **c** shows a CT axial lung window lesion located in a ground glass pulmonary nodule in the upper lobe of the right lung, with a tumor length of 1.5 cm. **d** shows a 100x pathological microscopic image of invasive adenocarcinoma with less dissemination in the STAS. **e**, **f** Male, 61. **e** shows a CT axial view of the lung window lesion located in a partial solid pulmonary nodule in the right upper lobe, with a CTR of 0.46. **f** shows a 100x pathological microscopic image of invasive adenocarcinoma with more dissemination in the STAS

Among all lesions, there were 57 solid nodules, of which 13 showed STAS. There were 60 subsolid nodules, of which four showed STAS (23.5%). Further subgroup analysis of subsolid nodules showed that among the 22 partially solid nodules, three showed STAS, and among the 38 ground-glass nodules, only one showed STAS (see Fig. 3). Solid nodules were more likely to show STAS than subsolid nodules. The incidence of STAS was highest in solid nodules, followed by partially solid nodules and then ground-glass nodules. The trend test results suggested that there was a statistically significant difference in STAS occurrence among the three groups (p for trend = 0.043). The chi-square test indicated that the incidence of STAS differed significantly among the three groups (Pearson chi-square = 7.49, *p* = 0.024), as shown in Table 2. Further pairwise comparisons revealed that the incidence of STAS in the partially solid nodule group was statistically different from that in the solid nodule group and the ground-glass nodule group.

**Table 2:** The chi-square test table for the incidence of STAS among different types of nodules

	STAS positive	STAS negative	Chi-square	p-value
Solid nodule, n (%)	13 (76.5)^b^	44 (44.0)^a^	7.49	0.024
Partial solid nodule, n (%)	3 (17.6)^a^	19 (19.0)^a^		
Ground-glass nodule, n (%)	1 (5.9)^b^	37 (37.0)^a^		

Each superscript letter indicates a subgroup of STAS categories, and at the significance level of *p* = 0.05, there is no significant difference in proportions between these subgroups. STAS refers to spread through air space

### The relationship between the primary tumor CTR of partially solid nodules and the risk of developing STAS

**Fig. 4:**
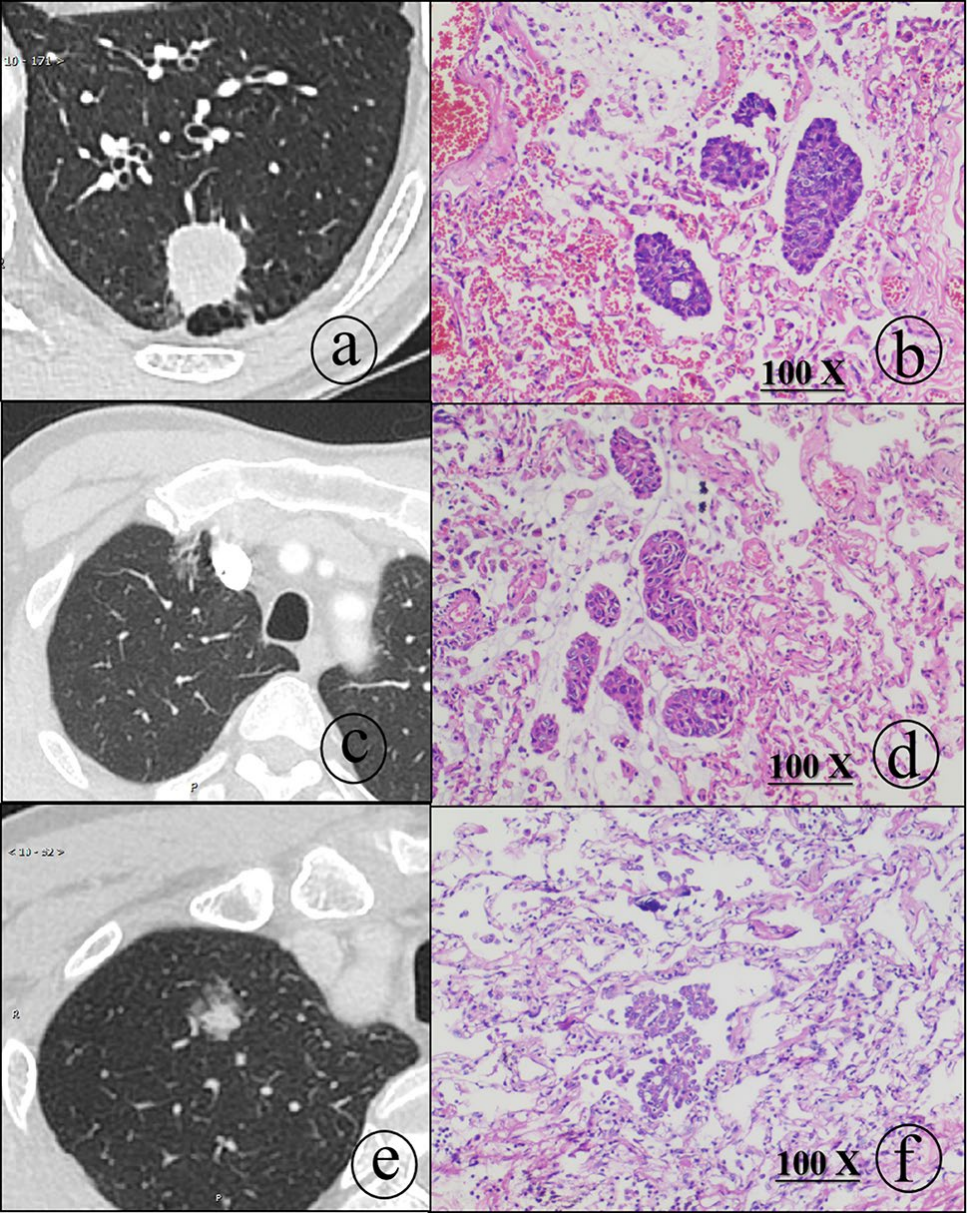
The smooth fitting curve between the CTR of the primary tumor and STAS. The smooth fitting curve showed that CTR tended to be linearly associated with STAS after adjusting for sex, age, BMI. The X-abscissa represented the CTR value, and the Y-ordinate represented the risk of primary tumor STAS. The solid black line represented CTR was linearly associated with STAS, and the black dotted lines above and below represent the 95% CI

A multiple regression model was constructed using the CTR of the primary tumor in partially solid nodules as the exposure variable and the risk of developing STAS as the outcome variable. Meanwhile, GAM (Fig. 4) was performed to visually assess the association between CTR and STAS after adjusting for sex, age, BMI, and pleural indentation, the smooth fitting curve showed that CTR tended to be linearly associated with STAS. The results indicated that as the CTR was increased, the risk of developing STAS in the primary tumor was also increased correspondingly. The regression model showed a *p*-value of 0.015 and an OR value of 1.24, indicating that for every one-unit increase in the CTR of the primary tumor in partially solid nodules, the risk of developing STAS was increased by 24%.

## Discussion

The main results of this study could be summarized as the three aspects as follows: In peripheral stage IA lung adenocarcinoma (1), pleural indentation of the primary tumor was independent risk factors for STAS following propensity score–matched analysis; (2) the incidence of STAS differed significantly between the group with partially solid nodules and those with solid or ground-glass nodules; and (3) as the proportion of solid components was increased in partially solid nodules, the risk of STAS in the lesion was also gradually increased.

STAS not only affects patient prognosis but also influences the choice of surgical approach. Previous studies [22, 23] have shown that, in stage I lung adenocarcinoma patients who undergo limited resection, compared to those who undergo lobectomy, the prognosis is worse when STAS is present. Therefore, assessing the risk of STAS in the primary tumor through preoperative CT imaging can provide diagnostic information in selecting surgical approaches, and can impact patient prognosis. In this study, the incidence of STAS was higher than that of occult lymph node metastasis (14.5% vs. 9.4%). Thus, stage IA lesions were more likely to develop STAS compared to occult lymph node metastasis. Furthermore, 10 cases (8.5%) of patients with STAS did not exhibit occult lymph node metastasis, which highlighted the need for thoracic surgeons to be particularly vigilant. Among patients with stage IA adenocarcinoma in this study, the incidence of STAS was found to be highest in solid nodules, followed by partially solid nodules and ground-glass nodules. while only one case of STAS (5.9%) was found in ground-glass nodules, with the remaining 16 cases found in solid and partially solid nodules. This is consistent with Kim’s [15] results. These findings suggested that the occurrence of STAS was closely related to the solid component of the nodule on CT. Therefore, the likelihood of STAS was increased correspondingly when nodules appeared as solid or partially solid on CT. This result might be explained as the solid component of the tumor was increased on CT, it indicated increased infiltration of tumor cells into the alveoli. STAS has also been shown to be a manifestation of tumor invasiveness. As infiltration increases, the risk of the tumor spreading to the bronchi or airways also increases. Thus, an increase in the solid component of a nodule implies an increase in tumor invasiveness and also indicates an increased risk of developing STAS. We will also try to determine the optimal diagnostic threshold for identifying high-risk STAS in partially solid nodules in the following research. Moreover, we confirmed that, after propensity score-matched analysis, the patients who did not have diabetes but had a history of pulmonary diseases, such as COPD, pulmonary fibrosis, emphysema, pulmonary bullae, chronic inflammation, and bronchiectasis, as well as those with spiculated and pleural indentation lesions, large size of solid components, solid nodule and high CTR subgroup were more likely to have STAS. Although the p values of the solid size of the primary tumor and the diabetes patients between the two groups were only 0.049 and 0.047, we believe that this is related to the small sample size. In addition, we also confirmed by propensity matching score analysis that the presence of pleural indentation in primary lesions was an independent risk factor for the STAS in peripheral stage IA lung adenocarcinoma. Therefore, the clinical predictive value of pleural indentation sign for STAS is crucial in selecting surgical methods. For stage IA lesions, when peripheral lung adenocarcinoma showed pleural indentation, thoracic surgeons should be aware of the risk of STAS when developing a surgical resection plan and choose an appropriate surgical approach.

Futhermore, in the CT characteristic analysis of the primary tumor, we also found that all 17 positive lesions showed bronchial abnormality, lobulation, and internal vascular sign. These findings suggested that if there were above-mentioned signs in the CT characteristics of the primary tumor, the likelihood of STAS was higher. Of course, this requires further verification by expanding the number of positive cases. Through the construction of a multiple regression model, we further confirmed that in partially solid nodules, the risk of STAS was increased by 24% for every unit increase in CTR. Therefore, for thoracic surgeons, measuring the CTR of partially solid nodules can help assess the risk of STAS and provide important diagnostic information for surgical procedure and patient prognosis evaluation. The results of this study suggested that peripheral stage IA partially solid nodules with a small CTR should be classified as low-risk nodules for invasiveness. In such cases, sublobar resection is recommended as the preferred surgical approach during surgical resection.

### Limitations

This study has several limitations. Firstly, it is a single-center retrospective study, which may have some selection bias, and lacks prospective validation. Secondly, the sample size needs to be further expanded, as there are only 17 positive cases, these cases are not classified according to the subtypes of bronchial abnormality and internal vascular signs. This work will be carried out as the number of positive cases accumulates. and the results of this study only apply to peripheral stage IA lung adenocarcinoma lesions. Additionally, this study has not yet determined the optimal diagnostic threshold for identifying high-risk STAS in partially solid nodules, which will be addressed in future studies.

## Conclusion

For patients with peripheral stage IA lung adenocarcinoma, pleural indentation of the primary tumor was independent risk factors for STAS, the risk of STAS was gradually increased with the increase of the solid component of the primary tumor. This can be used to assess the risk of STAS in peripheral stage IA lung adenocarcinoma patients and guide thoracic surgeons in surgical procedure.

## Data Availability

All data generated or analyzed during this study are available from the corresponding author upon reasonable request.

## Abbreviations

LDCT: low-dose computed tomography
NSCLC: Non-small-cell lung cancer
STAS: spread through air spaces
CTR: Consolidation tumor ratio
COPD: chronic obstructive pulmonary disease
SD: standard deviation
IQR: interquartile range
ICC: intraclass correlation coefficient
VIF: variance inflation factor
GAM: generalized additive model
OR: odds ratio
